# Advances in Twin-Screw Granulation

**DOI:** 10.3390/pharmaceutics14010046

**Published:** 2021-12-27

**Authors:** Valérie Vanhoorne, Ashish Kumar

**Affiliations:** 1 Laboratory of Pharmaceutical Technology, Department of Pharmaceutics, Faculty of Pharmaceutical Sciences, Ghent University, Ottergemsesteenweg, B-9000 Ghent, Belgium; 2 Pharmaceutical Engineering Research Group (PharmaEng), Department of Pharmaceutical Analysis, Faculty of Pharmaceutical Sciences, Ghent University, Ottergemsesteenweg, B-9000 Ghent, Belgium

**Keywords:** continuous manufacturing, wet granulation, pharmaceutical processing

Twin-screw granulation (TSG) is an emerging process technology that allows both wet and dry granulation of powders with a wide range of properties. This technology is of particular interest to the pharmaceutical industry, which is currently aiming to shift from batch to continuous mode manufacturing. TSG found its roots in the late 1990s, evolved rapidly in the last decade as a promising wet granulation technology and was commercialized by some of the leading equipment manufacturers, such as GEA and ThermoFisher. Drying is the obvious downstream step of twin-screw wet granulation and is achieved mainly by a semi-continuous fluid-bed dryer [1]. While the early applications of TSG were focused on the wet granulation process, it is now gaining ground as an alternative dry granulation method, namely melt granulation [2]. This evolution of technology is also evident in this Special Issue, where articles related to both wet and melt granulation have found a place.

While in initial papers on TSG, single placebo formulations were often used, focusing on process optimization, in recent years, various papers have addressed formulation optimization for TSG. The review paper of Portier et al. [3] summarized recent progress and opportunities in the formulation and equipment design on continuous TSG with special attention to formulation aspects. In general, formulations consist of an active pharmaceutical ingredient (API), filler (combination) and binder, at the least. Filler combinations of a soluble filler and microcrystalline cellulose (MCC), with the soluble filler/MCC ratio exceeding 1, are commonly used. APIs and fillers constitute the largest fraction of a formulation to be processed via TSG. Whereas the use of surfactants in formulations is not uncommon, binders are routinely added to formulations to favor the production of high-quality granules and tablets. Therefore, binder selection for TSG was addressed in two research papers [4,5]. As the residence time in the process is much shorter compared to batch-wise wet granulation processes, it was anticipated that critical binder characteristics could differ between continuous and batch-wise granulation techniques. In both studies, binary filler/binder mixtures were granulated in a filler/binder ratio of 95/5, and the binders were included in the powder mixture prior to granulation. Whereas Köster et al. [5] focused on the combined impact of binders and the specific feed load on granule size distributions and final tablet properties of a lactose-based formulation, Vandevivere et al. [4] focused on the critical binder attributes impacting the granule friability of both a mannitol- and dicalciumphosphate (DCP)-based formulation. The wettability of binder particles by water, as well as the wettability of surfaces by binder solutions and the low surface tension of binder solutions, proved crucial for both mannitol-based and DCP-based formulations. In contrast, different binder characteristics in terms of viscosity and dissolution kinetics were preferred for the mannitol- and DCP-based formulations, with high viscosity binders exhibiting slow dissolution kinetics preferred for DCP-based formulations. Mannitol, as a highly soluble filler exhibiting fast dissolution kinetics, contributed to bond formation during granulation. As a result, the added value of the inclusion of binders was limited, although at higher liquid-to-solid ratios, lower friability could be achieved by including a binder. However, this could not be generalized, as it was observed that by inclusion of, for example, hydroxypropyl methylcellulose (HPMC) E15 in the formulation, higher friabilities were observed compared to pure mannitol granules. Similarly, Köster et al. [5] demonstrated that inclusion of HPMC Pharmacoat 606 resulted in more fines in the lactose-based formulation compared to pure lactose granules. Furthermore, both studies highlighted a negative impact of binders on disintegration times, with some binders resulting in unacceptably long disintegration times [4,5]. Nevertheless, disintegrants were hypothesized to be able to compensate for the delay in disintegration due to the inclusion of a binder. Next to wet granulation, melt granulation using a continuous twin-screw granulator is of high interest as a versatile technique for the processing of moisture-sensitive APIs not requiring a drying step. It is applicable to various applications including immediate release, sustained release, taste-masking or solubilization. In melt granulation, binders are crucial in the formulation, as the heating of binders during the process is required for the softening or melting of the binders to create bridges between the particles of the formulation. Forster et al. [2] reviewed the excipients used in melt granulation aiming at various applications and highlighted the melting point or glass transition temperature of the binder and API, API/binder miscibility at the processing temperature and binder viscosity as critical formulation attributes. Moreover, the impact of critical process settings, including process temperature, screw design and barrel filling degree, were reviewed. Next to the research studies with formulation focus, equipment and process knowledge for TSG is also being developed through a combined experimental and theoretical (i.e., model-based) approach [6,7,8,9]. This is also quintessential because experiments are necessary to gather new knowledge about the system, whereas the theory is needed to put forth hypotheses to build further on this knowledge and gain fundamental understanding. Population balance models (PBMs) are extensively used for modeling the granulation processes. Since these models are semi-mechanistic and require adaptation to capture the physical processes within a TSG, an extensive experimental calibration using experimental datasets is done. Barrera Jiménez et al. [9] proposed improvement in an existing compartmental one-dimensional PBM for a TSG process by altering the original aggregation kernel in the wetting zone by applying an identifiability analysis. This resulted in a reduction in the number of model parameters to be calibrated, and these model parameters could be linked to the material properties and the liquid to solid ratio, which allows the creation of a generic PBM to predict the particle size distribution. Residence time distributions (RTDs) are another approach of gathering a thorough understanding of the process dynamics within TSG. While RTDs can be used to optimize the design space of the process, their measurement can be very challenging given the fast process. Muddu et al. [6] presents a new approach to estimate various residence time central moment metrics in a TSG, Mean residence time (MRT) and variance through a semi-mechanistic relations. The proposed approach was validated using different datasets in published literature. While the approach gave good results in some cases, lack of sufficient data to train the model was reflected in performance deficiency. A more mechanistic approach can be applied in the future to reduce the dependence on the excessive amount of data. Kumar et al. [7] applied a mechanistic approach to study the mixing of solids and liquids within the kneading zone of TSG using discrete element method (DEM). The study of the time evolution of particle flow and liquid distribution between particles in TSG revealed that agglomeration is a rather delayed process that takes place once the free liquid on the particle surface is well distributed. This understanding can be applied to design a more efficient TSG process and equipment by configuring the screw design as per the requirement of mixing. However, the findings in this study were preliminary, and a more extensive investigation of solid-liquid mixing along the complete length of a TSG should be performed before detailed understanding. Although a high computational demand restricted Kumar et al. [7] in performing these studies, since every particle in the simulation was tracked, the information regarding the velocity and contact frequency can be extracted to be used in PBMs for more mechanistic, yet still computationally realistic, simulations.

As a conclusion of this Special Issue, “Advances in Twin-Screw Granulation”, we trust that the published articles summarise the state-of-the-art in TSG and show some recent advances in the field. Moreover, the field is extending its scope and evolving to improve the existing technology and deliver to the unmet needs of continuous pharmaceutical manufacturing.
